# Partial germline reversions can increase VRC07 potency and breadth

**DOI:** 10.1186/1742-4690-9-S2-P101

**Published:** 2012-09-13

**Authors:** RS Rudicell, I Georgiev, Z Yang, S O'Dell, Y Kwon, J Zhu, X Wu, PD Kwong, JR Mascola, GJ Nabel

**Affiliations:** 1 Vaccine Research Center, NIAID, NIH, Bethesda, MD, MD, USA

## Background

VRC01 and related antibodies target the CD4 binding site (CD4bs), are broadly neutralizing and highly potent, and have undergone high levels of somatic hypermutation. To optimize such antibodies for passive immunization and to further understand antibody development, we reverted three CD4bs antibodies towards their putative germlines and analyzed the effects on breadth and potency. Interestingly, we also identified key germline reversion mutations that increased neutralization potential.

## Methods

Structure/function-based analyses were used to design partially reverted heavy and light chains based on the clonally-related antibodies VRC01, NIH45-46, and VRC07. Mature CDRs were maintained and framework regions were back-mutated. The antibodies were expressed, purified, and tested for binding to gp120 by ELISA. Neutralization against a panel of tier 2 HIV-1 pseudotyped viruses was determined for select antibodies.

## Results

The heavy chains of VRC01, NIH45-46, and VRC07 are 42%, 41%, and 44% somatically mutated from their germline precursor, while the light chains are 29% (VRC01/07) and 27% (NIH45-46) somatically mutated. We began by reverting over half of the heavy chain somatic mutations and over one-third of the light chain somatic mutations to their germline residue identities. An iterative design approach was used, and we systematically re-introduced mature residues to the partial germline reversions. Most mutants retained the ability to bind gp120 and neutralize diverse HIV-1 pseudoviruses, albeit with lower breadth and/or potency than their mature counterparts. Additionally, we found 3 partial-germline reversion mutations that increased VRC07 potency.

## Conclusion

Here, we showed that in most cases mature framework regions in addition to mature CDRs were required for highest neutralization potency and breadth. However, three framework germline reversion mutations increased potency 2-3 fold. These partial reversions are being combined with other mutations, including those that modulate Fc effector function, to optimize the antibody function for passive transfer in NHPs and humans.

